# Supplementary material for: A Photoluminescence Study of Eu3+, Tb3+, Ce3+ Emission in Doped Crystals of Strontium-Barium Fluoride Borate Solid Solution Ba4−xSr3+x(BO3)4−yF2+3y (BSBF)
Source: Materials (Basel). 2023 Jul 29;16(15):5344. doi: 10.3390/ma16155344 (PMC10419385; doi:10.3390/ma16155344)
Supplement: Supplementary file 1 [file materials-16-05344-s001.zip › materials-2454934-supplementary.pdf]

Article

A Photoluminescence Study of Eu^{3+}, Tb^{3+}, Ce^{3+} Emission in Doped Crystals of Strontium-Barium Fluoride Borate Solid Solution $Ba_{4-x}Sr_{3+x}(BO_3)_{4-y}F_{2+3y}$ (BSBF)

Tatyana B. Bekker [1,2,*], Alexey A. Ryadun [3], Sergey V. Rashchenko [1,2], Alexey V. Davydov [1,2], Elena B. Baykalova [2] and Vladimir P. Solntsev [1,2]

1 Sobolev Institute of Geology and Mineralogy, Siberian Branch of the Russian Academy of Sciences, 630090 Novosibirsk, Russia; rashchenkos@gmail.com (S.V.R.); davydov.av@gmail.com (A.V.D.); solntsev@igm.nsc.ru (V.P.S.)
2 Department of Geology and Geophysics, Novosibirsk State University, 630090 Novosibirsk, Russia; e.baikalova@nsu.ru
3 Nikolaev Institute of Inorganic Chemistry, Siberian Branch of the Russian Academy of Sciences, 630090 Novosibirsk, Russia; ryadunalexey@mail.ru
* Correspondence: t.b.bekker@gmail.com or bekker@igm.nsc.ru

Supporting Information

Table S1. Details of data collection and structure refinement for $Ba_{4-x}Sr_{3+x}(BO_3)_{4-y}F_{2+3y}$:$Eu^{3+}$ crystal.

Crystal data	
Chemical formula	B3.703662 Ba3.172464 F2.889012 O11.11099 Sr3.827532
M_r	1043.8
Crystal system, space group	Hexagonal, $P6_3mc$
Temperature (K)	293
a, c (Å)	10.8896(4), 6.96443(19)
V (Å^3)	715.22(4)
Z	2
Radiation type	Mo $K\alpha$
μ (mm^{-1})	22.838
Crystal size (mm)	0.1 × 0.05 × 0.05
Data collection	
Diffractometer	Esperanto-*CrysAlis PRO*-abstract goniometer imported esperanto images
Absorption correction	Multi-scan<br>*CrysAlis PRO* 1.171.42.49 (Rigaku Oxford Diffraction, 2022) Empirical absorption correction using spherical harmonics, implemented in SCALE3 ABSPACK scaling algorithm.
T_{min}, T_{max}	0.352, 1
No. of measured, independent and observed [$I > 3\sigma(I)$] reflections	23306, 1987, 1253
R_{int}	0.0875
$(\sin \theta/\lambda)_{max}$ (Å^{-1})	0.694
Refinement	
$R[F^2 > 2\sigma(F^2)]$, $wR(F^2)$	0.020, 0.051
No. of reflections	643
No. of parameters	52
$\Delta\rho_{max}$, $\Delta\rho_{min}$ (e Å^{-3})	0.75, -0.92

Table S2. Bond valence calculations according to Brese and O'Keeffe (1991) for Eu^{3+} substitution in Sr, Ba, and M sites in the $Ba_{4-x}Sr_{3+x}(BO_3)_{4-y}F_{2+3y}$ solid solution.

Bond	Bond length	Ligand occupancy	Bond valence (Eu^{3+})
Sr-X2O	2.390	0.47	0.20
Sr-X2F	2.450	0.27	0.07
Sr-X2F	2.450	0.27	0.07
Sr-O2	2.516	1.00	0.30
Sr-O2	2.516	1.00	0.30
Sr-X2F	2.520	0.27	0.06
Sr-X2F	2.520	0.27	0.06
Sr-X1F	2.617	0.30	0.05
Sr-O2	2.627	1.00	0.23
Sr-O2	2.627	1.00	0.23
Sr-X2O	2.730	0.47	0.08
Sr-O1	3.092	1.00	0.06
Sr-O1	3.093	1.00	0.06
**BVS(Eu^{3+})**			**1.78**
Ba-O1	2.700	1.00	0.19
Ba-F1	2.765	1.00	0.11
Ba-X2F	2.784	0.27	0.03
Ba-X2F	2.784	0.27	0.03
Ba-O1	2.853	1.00	0.12
Ba-O1	2.854	1.00	0.12
Ba-O2	2.926	1.00	0.10
Ba-O2	2.926	1.00	0.10
Ba-O2	3.015	1.00	0.08
Ba-O2	3.015	1.00	0.08
Ba-X2O	3.294	0.47	0.02
Ba-X2O	3.299	0.47	0.02
Ba-F1	3.517	1.00	0.01
Ba-X2F	3.714	0.27	0.00
Ba-X2F	3.714	0.27	0.00
**BVS(Eu^{3+})**			**1.01**
M-X2F	2.590	0.27	0.05
M-X2F	2.590	0.27	0.05
M-X2F	2.590	0.27	0.05
M-X2F	2.590	0.27	0.05
M-X2F	2.590	0.27	0.05
M-X2F	2.590	0.27	0.05
M-X2O	2.590	0.47	0.12
M-X2O	2.590	0.47	0.12
M-X2O	2.590	0.47	0.12
M-X1F	2.700	0.30	0.04
M-O2	2.806	1.00	0.14
M-O2	2.806	1.00	0.14
M-O2	2.806	1.00	0.14
M-O2	2.806	1.00	0.14
M-O2	2.806	1.00	0.14
M-O2	2.806	1.00	0.14
**BVS(Eu^{3+})**			**1.52**

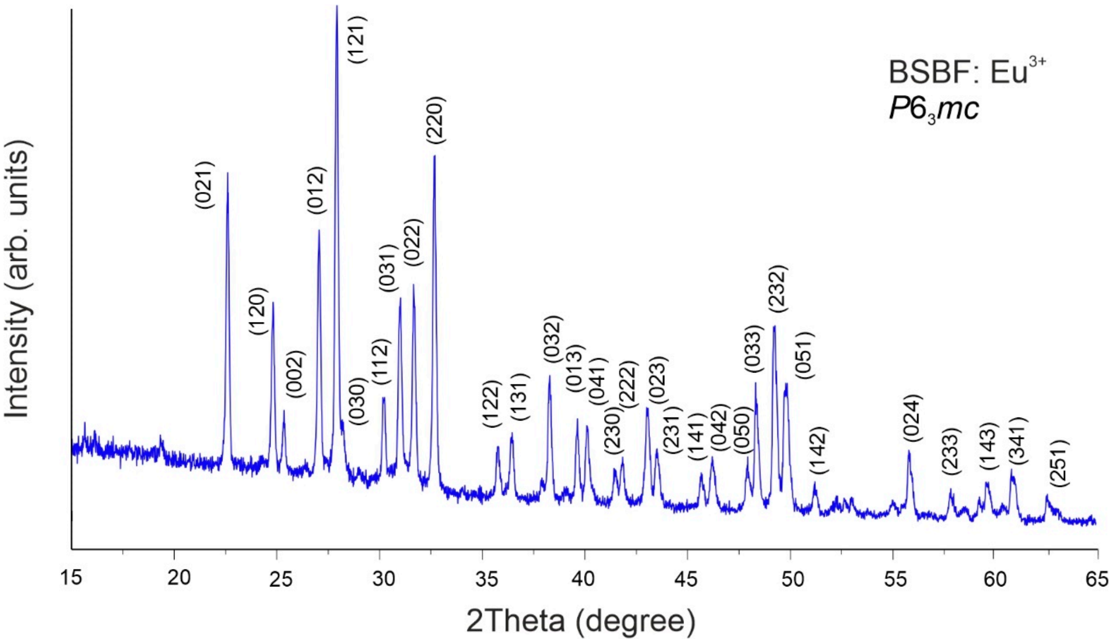

Figure S1. X-ray powder diffraction pattern of the BSBF: Eu^{3+} crystal.
